# Editorial: Novel drug discovery and design in acute myeloid leukemia: from bench to bedside

**DOI:** 10.3389/fonc.2024.1449636

**Published:** 2024-10-18

**Authors:** Yan-Lai Tang, Zhenhua Chen, Xue-Qun Luo

**Affiliations:** ^1^ Department of Pediatrics, The First Affiliated Hospital, Sun Yat-sen University, Guangzhou, Guangdong, China; ^2^ Department of Systems Biology, Beckman Research Institute, City of Hope, Duarte, CA, United States

**Keywords:** acute myeloid leukemia (AML), CRISPR-Cas9, ATO, precision medicine, genomic and functional traits

The treatment of acute myeloid leukemia (AML) continues to pose significant challenges, primarily attributed to its genetic diversity and intricate pathophysiological mechanisms. Recent developments in genomic and functional precision medicine have brought about innovative approaches to drug discovery and design in the treatment of acute myeloid leukemia, offering potential benefits for enhancing patient outcomes.

In this Research Topic, there are five articles (three original research articles and two review articles) published, focusing on recent advancements in genomic and functional precision medicine.

The CRISPR-Cas9 screening technique has emerged as a powerful method for identifying drug sensitivity at the genetic level. By using CRISPR-Cas-9, the study confirmed that multi-target anticancer drug ATO’s sensitivity is controlled by oxidative stress, metabolism, chemokine, cytokine, and immune responses. (Chen et al.). It was shown that patients with acute myeloid leukemia (AML) who expressed more KEAP1 had a worse overall survival (OS). KEAP1 is a pivotal gene in ATO drug sensitivity. This gene could be targeted to improve the efficacy of ATO in treating various cancers. (Chen et al.). This comprehensive study using genome-wide CRISPR-Cas9 screening provides insights into the genetic and molecular mechanisms regulating ATO sensitivity. These findings pave the way for improved cancer treatments by targeting identified pathways and genes to overcome ATO resistance.

Autophagy is a cellular process that degrades damaged organelles and proteins, playing a regulatory role in various hematologic malignancies, including AML. From the Web of Science Core Collection, Gao et al. retrieved literature on autophagy and AML from 2003 to 2023.A total of 343 articles were published in 169 journals, authored by researchers from 295 institutions across 43 countries. Research hotspots in AML include genetic regulation, autophagy inhibition, and targeted drugs and future trends point to chemotherapy resistance and mitochondrial autophagy.

Patients with AML who are unfit for intensive chemotherapy are commonly treated with low-dose cytarabine (LDAC). Although LDAC has shown the capacity to induce differentiation *in vitro*, this effect is infrequently observed *in vivo*. (Smoljo et al.). The presence of BM stromal cells inhibits LDAC-induced differentiation of AML cells, which likely contributes to the limited differentiation observed *in vivo* in AML patients treated with LDAC. The study provides valuable insights into the role of the BM microenvironment in AML therapy and suggests potential strategies for enhancing differentiation-based treatments.

Treating relapsed or refractory acute myeloid leukemia (R/R AML) and myeloid sarcoma (MS) presents significant challenges. Tong et al. evaluate the efficacy and safety of selinexor-containing chemotherapy-free or low-dose chemotherapy regimens for patients with R/R AML and MS. The study finds that these selinexor-containing regimens are feasible, tolerable, and offer a viable opportunity for transplantation in R/R AML and MS patients.

In recent years, functional and genomic approaches have been implemented to guide targeted therapy for AML patients. Bhatia et al. conclude that integrating genomic and functional precision medicine is crucial for advancing AML treatment. Despite progress in identifying genetic mutations and developing targeted therapies, challenges such as low response rates and the complexity of AML’s genetic landscape persist. The review explores future strategies, emphasizing the necessity of combining genomic and functional approaches to enhance precision medicine in AML treatment.

The papers collected in this Research Topic show recent progress and potential future research trends in the discovery and design of novel drugs for AML. Chen et al. summarized genomic and functional therapeutic biomarkers adopted for AML therapy. Gao et al. highlighted current research hotspots, including genetic regulation, autophagy inhibition, and autophagy-related targeted drugs. Smoljo et al. demonstrates that BM stroma reduces differentiation of AML induced by LDAC. Tong et al. concluded that chemotherapy-free or low-dose chemotherapy regimens combined with selinexor for R/R AML are feasible and tolerable. Bhatia et al. summarized genomic and functional therapeutic biomarkers for AML therapy, and discussed the challenges associated with these approaches. The studies reviewed in this editorial present a promising frontier in AML treatment.

